# Genome‐wide association analysis for response of Senegalese sorghum accessions to Texas isolates of anthracnose

**DOI:** 10.1002/tpg2.20097

**Published:** 2021-04-26

**Authors:** Ezekiel Ahn, Louis K. Prom, Zhenbin Hu, Gary Odvody, Clint Magill

**Affiliations:** ^1^ Dep. of Plant Pathology & Microbiology Texas A&M Univ. College Station TX 77843 USA; ^2^ USDA‐ARS Southern Plains Agricultural Research Center College Station TX 77845 USA; ^3^ Donald Danforth Plant Science Center Saint Louis MO 63132 USA; ^4^ Texas A&M AgriLife Research Corpus Christi TX 78406 USA

## Abstract

Anthracnose disease of sorghum is caused by *Colletotrichum sublineola*, a filamentous fungus. The genetic basis of resistance to anthracnose in sorghum is largely unclear, especially in Senegalese sorghum germplasm. In this study, 163 Senegalese sorghum accessions were evaluated for response to *C*. *sublineola*, and a genome‐wide association study (GWAS) was performed to identify genetic variation associated with response to *C. sublineola* using 193,727 single nucleotide polymorphisms (SNPs) throughout the genome. Germplasm diversity analysis showed low genetic diversity and slow linkage disequilibrium (LD) decay among the Senegalese accessions. Phenotypic analysis resulted in relatively low differences to *C. sublineola* among the tested population. Genome‐wide association study did not identify any significant association based on a strict threshold for the number of SNPs available. However, individual analysis of the top eight SNPs associated with relative susceptibility and resistance identified candidate genes that have been shown to play important roles in plant stress tolerance in previous studies. This study identifies sorghum genes whose annotated properties have known roles in host defense and thus identify them as candidates for use in breeding for resistance to anthracnose.

## Core Ideas

- A total of 163 Senegalese sorghum accessions were evaluated for response to *C. sublineola*.
- A genome‐wide association study was performed to identify genetic variation associated with response to *C. sublineola*.
- Top single‐nucleotide polymorphisms were associated with genes known to contribute to defense responses.

**Abbreviations**
APE: Analysis of Phylogenetic and Evolution
GBS: genotype‐by‐sequencing
GWAS: Genome‐Wide Association Study
Hpa: *Hyaloperonospora arabidopsidis*
LD: linkage disequilibrium
LRR: leucine rich repeat
NPGS: National Plant Germplasm System
PC: principal component
PCA: principal components analysis
SAP: sorghum association panel
SAT: semi‐arid tropics
SNP: single‐nucleotide polymorphism.

## 1 INTRODUCTION

Sorghum [*Sorghum bicolor* (L.) Moench] is one of the most important cereal crops grown in the semiarid tropics (SAT) of Africa, Asia, Australia, and the Americas for its food, feed, fodder, and fuel value (Ashok Kumar, 2019). Although impressive progress has been made in increasing productivity over recent years, global sorghum production is still constrained by several biotic and abiotic stresses (Ashok Kumar, 2019). Anthracnose caused by *Colletotrichum sublineola* Henn. ex Sacc. & Trotter 1913 is an important disease of cultivated sorghum worldwide (Xavier et al., 2018) and thus has been the object of several studies attempting to reveal genes associated with disease response. Cuevas et al. (2018a) reported genes that encode an F‐box, protein tyrosine kinase, leucine rich repeat, oryzalide A biosynthesis, glucuronosyl transferases, and peroxidase as top candidate defense related genes in response to *C. sublineola* inoculation using genome‐wide association study (GWAS) with a set of 377 diverse sorghum cultivars assembled to create sorghum association panel (SAP) lines for use in association mapping (Casa et al., 2008). Prom et al. (2019) reported genes whose products include motifs such as pentatricopeptide repeat, leucine rich repeat, 9‐cis‐epoxycarotenoid dioxygenase, and calcium‐binding EGF domain genes as top candidates in the same SAP lines, also in response to *C. sublineola* but in a different environment and with different isolates. Ahn et al. (2019) reported genes that encode proteins with a zinc finger domain, an F‐box domain, exodeoxyribonuclease VII, and more as top candidate defense related genes associated with resistance in a sorghum mini core collection that also assembled to maximize diversity (Upadhyaya et al., 2009). In that study, in addition to response to anthracnose, GWAS analysis also sought markers associated with two other diseases, downy mildew and head smut, which are caused by the pathogens *Peronosclerospora sorghi* and *Sporisorium reilianum*, respectively (Ahn et al., 2019). Different sorghum lines provide different gene pools, and as a result, these studies identified potential candidate defense related genes against *C. sublineola* in each population. Multiple genes and gene combinations contribute to host responses to a pathogen, which in the case of *C. sublineola* is also known to have many pathotypes as defined by tests on hosts with different resistance genes (Prom et al., 2012).

Sorghum germplasm from West and Central Africa is an important source of resistance genes to fungal diseases (Cuevas et al., 2018b). In a recent study, Cuevas et al. (2018b) evaluated Senegalese sorghum accessions for agronomic traits, anthracnose, and grain mold resistance at the Isabela and Mayaguez experimental farm in Puerto Rico. In another study, Faye et al. (2019) conducted a GWAS based on sorghum landrace accessions from Senegal and adjacent locations to better characterize variation underlying putative adaptive traits including photoperiodic flowering and inflorescence morphology.

It is hypothesized that Senegalese sorghum accessions would respond differently to Texas anthracnose isolates compared with those used in the study in Puerto Rico (Cuevas et al., 2018b) due to different pathotypes of *C. sublineola* and environmental conditions. In the current study, 163 sorghum Senegalese accessions, including those tested in Puerto Rico, were scored for responses to Texas isolates of *C. sublineola* inoculation in a greenhouse located in College Station, TX. Part of the rationale was to compare the disease response to Texas isolates compared with those from Puerto Rico. The disease response results were combined with a GWAS analysis that includes 193,727 single‐nucleotide polymorphic (SNP) loci from a publicly available genotype‐by‐sequencing (GBS) dataset for the Senegalese accessions. Results were generated using the GAPIT genome association and prediction integrated tool in R to identify chromosomal locations associated with differences in disease response.

When the top‐scoring SNPs were mapped to the published sorghum genome, in all cases, the nearest annotated gene had precedence for a role in host defense or stress response.

**Core Ideas**

- A total of 163 Senegalese sorghum accessions were evaluated for response to *C. sublineola*.
- A genome‐wide association study was performed to identify genetic variation associated with response to *C. sublineola*.
- Top single‐nucleotide polymorphisms were associated with genes known to contribute to defense responses.

## 2 MATERIAL AND METHODS

### 2.1 Sorghum lines

The 163 accessions (listed in Supplemental Table S1) were obtained from the USDA‐ARS Plant Genetic Resources Conservation Unit for this research.

### 2.2 Disease evaluations

The anthracnose evaluations were conducted in 2020 in a greenhouse at College Station, TX. Seeds from each accession, including BTx623, TAM428, and PI609251 (susceptible) and SC748‐5 (resistant) checks were planted at a rate of eight seeds per tall treepots (10 by 35 cm, 4 by 14 inches) (Hummert International) containing Pro‐line 21 G potting mix (BWI,) mixed at a rate of 50:1 grams of osmocote classic fertilizer 17–7–12 (O.M. Scott & Sons Company). Four tall treepots were placed in 11.4‐L (3‐gallon) poly‐tainer cans (25 by 24 by 22 cm, 10 by 9.5 by 8.6 inches) (Hummert International) to maximize space in the greenhouse. At the three‐leaf stage, plants were thinned to four plants per pot. Each accession was replicated three times in a randomized complete block design. The greenhouse is constructed with plexiglass, which allows for natural light to pass through. The temperature in the greenhouse was set at 25 ± 2 ˚C. Inoculum was prepared by inoculating autoclaved sorghum grain with a spore suspension from a mix of eight local isolates of *C. sublineola* (FSP2, FSP5, FSP7, FSP35, and FSP36 from sorghum line BTx635, FSP46 and FSP50 from RTx2536, and FSP53 from BSBC) that were harvested after growth on half‐strength potato dextrose agar. These were the most virulent isolates based on the response of susceptible checks BTx623, TAM428, and RTx430 in field evaluation (Prom et al., 2019). The mixture of these isolates was used to represent the different pathotypes that may exist in the Texas A&M AgriLife Research Farm, Burleson County, Texas.

Inoculum preparation and inoculation methods were as previously described by Prom et al (2009). For inoculations using infested grain, sorghum seeds were place in a steel pan and soaked with tap water for 2 d. The water was drained and the pan with the seeds was autoclaved at 121 ˚C for 30 min. After cooling, agar plugs containing the five isolates were placed in the pan and incubated at 25 ˚C for 10 d. The contents of the pan were mixed every 2 d to allow for complete colonization of the seeds. At the 8‐to‐10‐leaf stage, 10 *C. sublineola*‐colonized sorghum seeds were dropped into the leaf whorl of each plant. For spray inoculations, each isolate was cultured separately in petri plates containing half‐strength potato dextrose agar. After 10 d of incubation, plates were flooded with sterile water and the spores dislodged using a rubber spatula. The suspensions were mixed in an Erlenmeyer flask and concentrate adjusted to 1 × 10^6^ spores ml^–1^. Plants with growth stage of 3 (eight‐leaf stage) were sprayed with the spore suspension until runoff. Following inoculation, plants were misted for 20 s at 1‐h intervals for 8 h d^–1^ for 14 d to provide a conducive condition for infection and disease development. Disease assessments were conducted 30 d after inoculating the plants. Disease ratings were based on a scale of 1–5 (Prom et al., 2009), where 1 = no symptoms or chlorotic flecks on leaves, 2 = hypersensitive reaction on the leaves but no acervuli formation, 3 = lesions with acervuli in the centers on the bottom leaves, 4 = necrotic lesions with acervuli on the bottom and middle leaves, and 5 = most leaves dead and infection on the flag leaf containing abundant acervuli. The symptom types were then categorized into two reaction types: ratings 1 or 2 as resistant; ratings 3, 4, or 5 as susceptible.

### 2.3 Genotyping data

Single‐nucleotide polymorphism data were extracted from an integrated sorghum SNPs dataset based on sorghum reference genome version 3.1.1 and originally genotyped using GBS (Elshire et al., 2011; Hu et al., 2019; Upadhyaya et al., 2013; Wang et al., 2013). Missing data were imputed using Beagle 4.1 (Browning, & Browning, 2016).

### 2.4 Population genomic analysis

The nucleotide diversity was analyzed using VCFtools with a 100‐kb sliding window (Danecek et al., 2009). Linkage disequilibrium (LD) decay was examined using PopLDdecay with ‐MaxDist 600, ‐MAF 0.05, ‐OutStat LD05 (Zhang et al., 2019); the LD decay figure was drawn with a custom R script. The principal components analysis (PCA) was performed using the SNPRelate package with maf = 0.05, missing.rate = 0.2 (Zheng et al., 2012). The phylogenetic tree was constructed using SNPRelate and visualized using the Analysis of Phylogenetic and Evolution (APE) package (Paradis et al., 2004).

### 2.5 GWAS and SNP mapping

Genome‐wide association study was performed using a linear mixed model in GAPIT with Model.selection = T, SNP.MAF = 0.01 (Lipka et al., 2012; Yu et al., 2005). The manhattan plots were made using the qqman package (Turner, 2014). Single‐nucleotide polymorphisms with high probability (*p* value <.0003) of contribution to disease responses were tracked to the specific chromosome location based on the sorghum reference genome sequence, version 3.1.1 available at the Phytozome 12 (https://phytozome.jgi.doe.gov https://phytozome.jgi.doe.gov), as updated in 2018 (McCormick et al., 2018).

## 3 RESULTS AND DISCUSSION

### 3.1 Phenotypic variation

**FIGURE 1:**
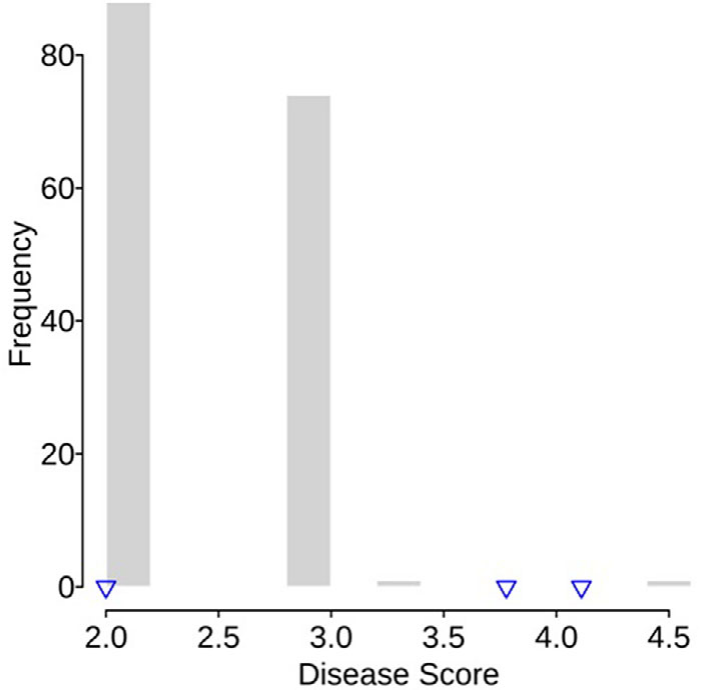
The phenotype distribution. The three blue triangles are the three checks. *X* axis indicates average scores based on disease evaluations, and *Y* axis is numbers of sorghum lines. Senegalese sorghum germplasm was scored relatively low and was skewed toward resistance with an average 2.47

To characterize the variation of anthracnose response of the Senegalese sorghum collection, each accession was evaluated in the greenhouse with three replicates, and the scores for the three replicates were highly correlated, with an average *r* ^2^ = .92. The rating values for the accessions ranged from 2.00 to 4.56 with an average 2.47 (Figure 1). The high susceptibility of the susceptible checks BTx623, PI609251, and TAM428 showed clear infections, whereas SC748‐5, the resistant check, did not show any infection, which indicates the inoculum was effective in identifying resistant accessions. The results showed that the range in susceptibility of the Senegalese sorghum germplasm was relatively low and was skewed toward resistance, with a majority of disease ratings below 3. Out of the 163 accessions tested, 88 accessions were resistant to *C*. *sublineola*, indicating that the Senegalese collection is an excellent source for anthracnose resistance. Using the same Senegalese collection from the U. S. National Plant Germplasm System (NPGS), Cuevas et al. (2018b) also evaluated 158 accessions for resistance in Isabela and Mayaguez, Puerto Rico using a single isolate of the anthracnose pathogen from each site as inoculum. Authors had noted that out of the 158 accessions, 77 were resistant when evaluated in Puerto Rico. Table 1 shows the reaction of 73 of those resistant accessions from Puerto Rico when evaluated with a mixture of 8 Texas isolates. A total of 29 resistant accessions in Puerto Rico were susceptible in Texas; however, 44 accessions were resistant when using isolates from both locations, indicating that these resistant accessions may possess stable resistance to geographically different anthracnose pathogens. In addition, some of the accessions in the current study, including PI514280, PI514287, PI514291, PI514300, and PI514322, were resistant when evaluated with the Texas isolates but susceptible in Puerto Rico (Cuevas et al., 2018a). *Colletotrichum sublineola* is a hyper‐variable pathogen and several pathotypes have been described in different sorghum growing regions using different differentials (Cardwell et al., 1989; Pande et al., 1991; Valèrio et al., 2005; Moore et al., 2008; Prom et al., 2012). Prom et al. (2012) noted that pathotypes from Texas are different from those identified in Puerto Rico, and the current study (Table 1) confirmed that. Similar to this result, it has been reported that sorghum SAP lines responded differently to anthracnose based on inoculum and field locations (Cuevas et al., 2018a). However, the sorghum accessions classified as resistant in Puerto Rico were also resistant when screened at Georgia and Texas, USA (Cuevas et al., 2018a; Cuevas & Prom 2020; Prom et al., 2019). Hence, it is not surprising to observe some of the Senegalese sorghum accessions inoculated with either Texas or Puerto Rico isolates being resistant in both locations.

**TABLE 1:** Reaction of the Senegalese accessions inoculated with *Colletotrichum sublineola* isolates from Texas in the greenhouse conducted in 2019 compared with the published field inoculations of the same accessions inoculated with isolates from Isabela and Mayaguez, in Puerto Rico between 2014 and 2015

Accession	College Station	Puerto Rico	Accession	College Station	Puerto Rico
PI 514289	S	R	PI 514375	R	R
PI 514290	R	R	PI 514376	R	R
PI 514292	R	R	PI 514378	S	R
PI 514295	R	R	PI 514381	R	R
PI 514296	S	R	PI 514383	R	R
PI 514297	R	R	PI 514387	R	R
PI 514299	R	R	PI 514388	S	R
PI 514301	R	R	PI 514392	S	R
PI 514302	R	R	PI 514397	R	R
PI 514303	R	R	PI 514399	S	R
PI 514306	S	R	PI 514401	R	R
PI 514307	R	R	PI 514411	S	R
PI 514309	S	R	PI 514417	S	R
PI 514310	S	R	PI 514418	R	R
PI 514312	S	R	PI 514424	S	R
PI 514313	S	R	PI 514425	R	R
PI 514314	R	R	PI 514426	R	R
PI 514317	R	R	PI 514428	R	R
PI 514320	R	R	PI 514429	R	R
PI 514325	S	R	PI 514431	R	R
PI 514326	S	R	PI 514434	R	R
PI 514332	S	R	PI 514436	R	R
PI 514335	R	R	PI 514437	S	R
PI 514336	S	R	PI 514439	R	R
PI 514347	S	R	PI 514444	S	R
PI 514348	R	R	PI 514446	S	R
PI 514349	S	R	PI 514448	R	R
PI 514353	S	R	PI 514449	R	R
PI 514354	R	R	PI 514452	R	R
PI 514356	S	R	PI 514453	R	R
PI 514360	S	R	PI 514465	S	R
PI 514361	S	R	PI 514466	R	R
PI 514362	R	R	PI 514468	R	R
PI 514364	R	R	PI 514472	R	R
PI 514366	R	R	PI 514474	R	R
PI 514373	R	R	PI 514478	R	R
PI 514374	S	R	PI 514280	R	S
PI 514287	R	S	PI 514291	R	S
PI 514300	R	S	PI 514322	R	S

*Note*. Nearly identical Senegalese accessions that were screened in Isabela and Mayaguez, Puerto Rico by Cuevas et al., 2018b. The results of Puerto Rico study are from Cuevas et al., 2018b.

### 3.2 Population genomic diversity of the panel

**FIGURE 2:**
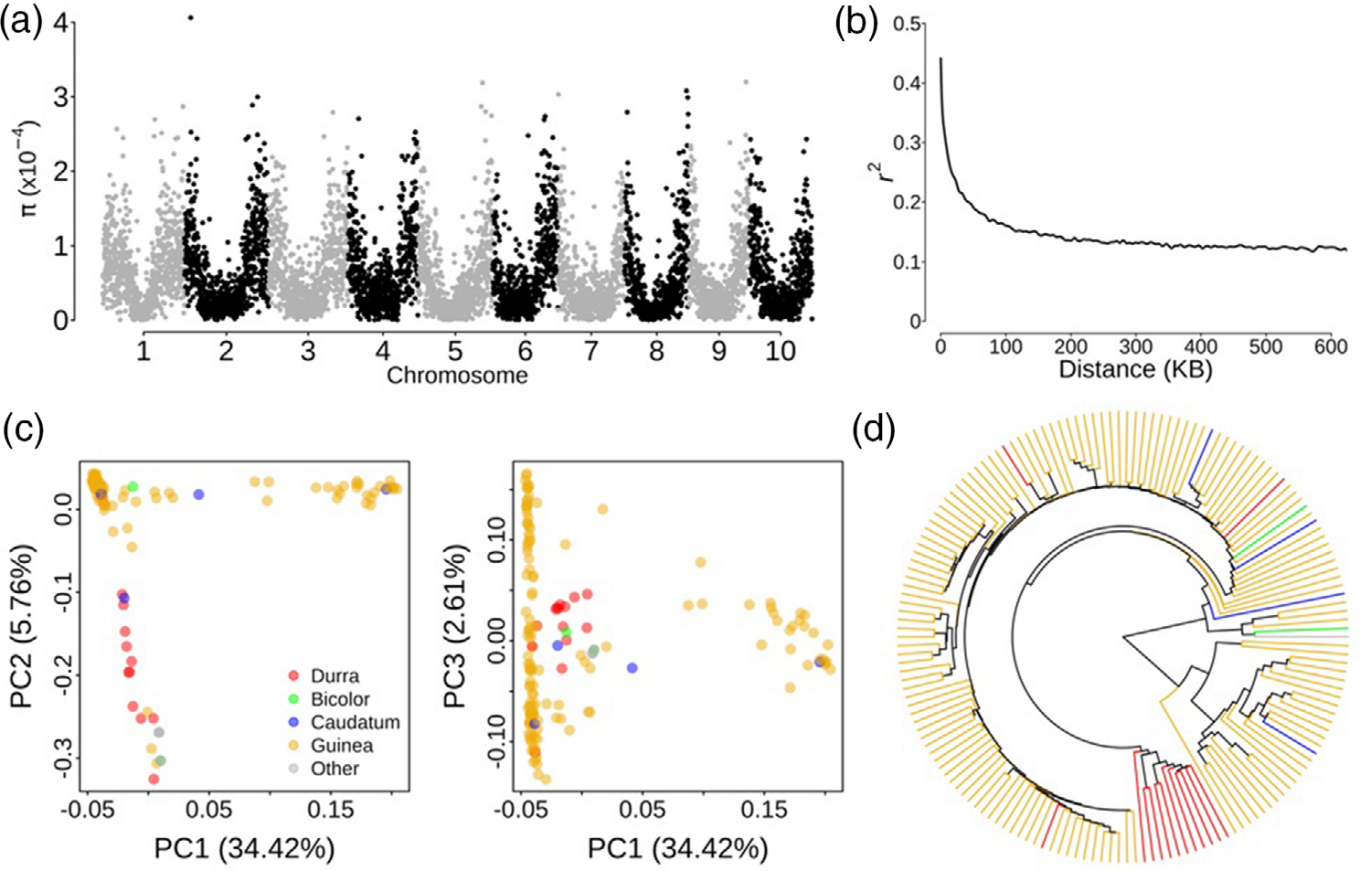
The population genomic diversity of the Senegal sorghum panel. (a) The nucleotide diversity with the 100 kb sliding window across the genome. (b) The LD decay. (c) The principal component analysis of Senegal sorghum panels, the colors indicate the different sorghum botanical races. (d) The phylogenetic tree of the Senegal sorghum accessions, the same color scheme as in part (c) relative to the botanical races of sorghum

The average nucleotide diversity (π) was 5.9 × 10^−5^, and the nucleotide diversity varied across the genome (Figure 2a), which was much lower as compared to that of the previous studies with (π) = 3.7 × 10^−4^ and 5.4 × 10^−4^ in durra & 6.0 × 10^−4^ in guinea accessions (Morris et al., 2013; Faye et al., 2019). The lower diversity may result from the small collections, especially when limited to accessions from the same country. The LD analysis showed that the LD decay distance was 800 bp when LD decay reached half the maximum value (42,500 bp at *r^2^* = .2) (Figure 2b).

The population structure was analyzed using principal component (PC) analysis. The first 20 PCs captured 58.92% variation, and the first PC explained 34.42% of the variation, which mainly explained the variation within accessions classified as race *Guinea* (Figure 2c). The second PC explained 5.76% of the variation, and the variation was mainly caused by the different botanical races (Figure 2a). The third PC mainly captured variation in race *Guinea* lines that was also captured by the first PC (Figure 2c). The phylogenetic tree showed similar result as the PCA, showing two major clusters within *Guinea* (Figure 2d) and another cluster for *Durra*. The population structure analysis is consistent with the previous studies that the botanical subrace was the major factor for the population diversity pattern (Morris et al., 2013; Hu et al., 2019). According to de Wet et al. (1972), sorghum was domesticated in Africa, with selection from the original *S. bicolor* giving rise to five distinct races as defined primarily by spikelet and grain morphology. Although the races are interfertile, local adaptations based on environmental factors affecting productivity and grower preferences resulted in a high degree of race maintenance, with accessions of races *Guinea* and *Durra* being most common in West Africa, including Senegal.

### 3.3 Genome‐wide association study

**FIGURE 3:**
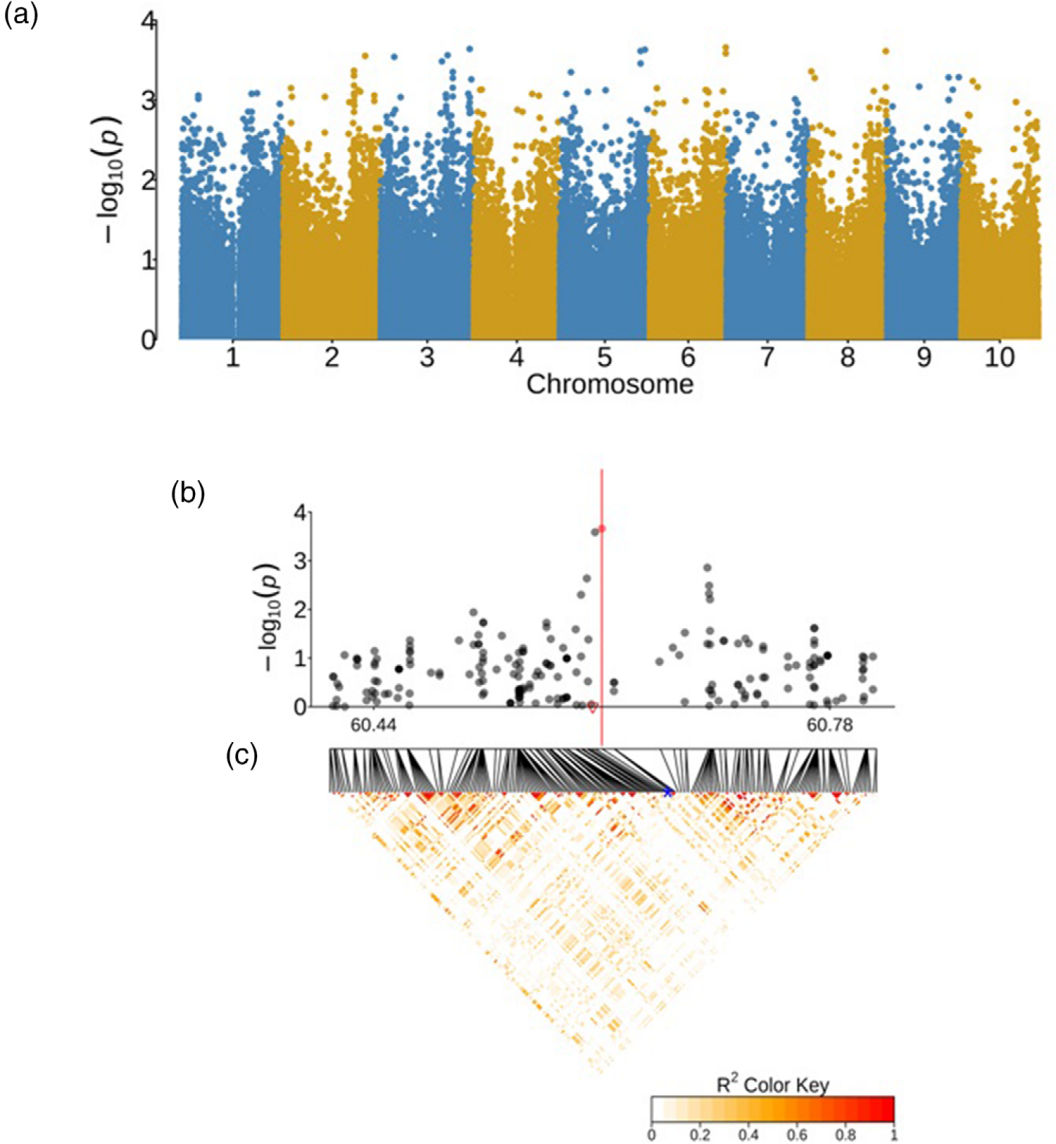
The genome‐wide association for response to anthracnose in sorghum. (a) The regional association on chromosome 6 around 200 kb of S06_60609133. (b) The regional associations at around 200 kb of the leading SNP S06_60609133. The leading SNP is highlighted by the red dot, the red triangle is the candidate; (c) LD heatmap of the association region around 200 kb of S06_60609133. The blue star is the leading SNP as shown in part (b)

There were no associations identified that reached the Bonferroni threshold, so the top eight associations were further analyzed based on the reference genome annotation and local LD landscape. We found eight high confidence candidates close to the associated SNPs (Figure 3 and Table 2).

**TABLE 2:** Annotated genes nearest to the most significant SNPs associated with anthracnose. The distance in base pairs to the nearest genes and *p* value are listed

Chr.	Location	Candidate gene	Associated annotation	Base pairs	*P* value
6	60609133	Sobic.006G274866	Leucine Rich Repeat // Protein tyrosine kinase // Leucine rich repeat N‐terminal domain	4,656	.000219
3	70974745	Sobic.003G401200	Selenium binding protein	15	.000229
5	67841876	Sobic.005G194700	Zinc finger, C3HC4 type family protein	0	.000234
5	64406687	Sobic.005G166700	Sulfotransferase family	5,538	.000243
8	61651261	Sobic.008G183100	Protein of unknown function// Domain of unknown function	0	.000244
3	53329242	Sobic.003G203500	Cytosolic aldehyde dehydrogenase RF2C Aldehyde dehydrogenase family	1,983	.000274
2	65282291	Sobic.002G268900	Single strand DNA repair‐like protein	0	.00028
3	10745341	Sobic.003G118600	F‐box domain	10,377	.000288

*Note*. Chr., chromosome; SNP, single‐nucleotide polymorphism.

When mapped back to the published genome, top candidates SNPs with *p* < .0003 were nearest to genes that have previously reported roles in biotic or abiotic resistance/stress responses (Figure 3 and Table 2).

Single‐nucleotide polymorphisms loci S06_60609133 and S06_60604148 tag the same gene(s) as both are located in or very near leucine rich repeat (LRR1)/protein tyrosine kinase (Sobic.006G274866). Leucine rich repeats are a feature of nearly all cloned resistance genes and are widely known for roles in plant host defense. The LRR is assumed to interact with another protein, which in the case of disease resistance triggers a signal transduction pathway to activate active host defense (Kourelis & van der Hoorn, 2018). As examples, silencing LRR1/PR4b in pepper (*Capsicum annuum* L.) and PR4b over‐expression in *Arabidopsis thaliana* demonstrated that LRR1 and PR4b are necessary for defense responses to *Pseudomonas syringae* pv. tomato and *Hyaloperonospora arabidopsidis* (Hpa) infection, respectively (Hwang et al., 2014). Similarly, silencing of an LRR1 gene was found to compromise resistance to the bacterial pathogen *Xanthomonas oryzae* pv. *oryzae* in rice (*Oryza sativa* L.) (Caddell et al., 2017). In GWAS studies of sorghum mini core collection and SAP lines, regarding anthracnose and head smut, leucine rich repeat containing proteins were listed as top candidate genes associated with response to anthracnose (Ahn et al., 2019; Cuevas et al., 2018a; Prom et al., 2019). The SNP locus S03_70974745 is 15 bp away from a protein similar to selenium binding protein. Selenium binding protein 1 (*SBP1*) gene expression studies in *Arabidopsis* revealed that it was increased when seedlings were challenged with several stresses such as sulfur deficiency (Hugouvieux et al., 2009). Similarly, selenium‐binding protein was induced by defense‐related signaling molecules methyl jasmonate and ethylene in *Arabidopsis* (Schenk et al., 2000). Constitutive expression of selenium‐binding rice gene *OsSBP* in rice was shown to enhance resistance to both blast and bacterial blight (Sawada et al., 2004).

The SNP locus S05_67841876 is located within the gene coding for a zinc finger family protein. Zinc finger containing proteins seem to be primarily involved in abiotic stress responses, but a number of zinc finger domains have been associated with disease resistance as components of previously cloned disease R genes in rice, tomato (*Lycopersicon esculentum* L.), and other plants (Gupta et al., 2012). Further, when the gene identified here was used in a BLAST search, results further identified the gene as coding a E3 ubiquitin‐protein ligase. These proteins have been shown to be critically involved in innate immunity in plants since they are involved in steps starting with pathogen perception and in regulating signaling of downstream events leading to the hypersensitive response (Delauré et al., 2008; Duplan & Rivas 2014). Thus, it is not surprising that other GWAS studies have identified zinc finger proteins as top candidate resistance genes for sorghum mini core collection and SAP lines against *C. sublineola* (Ahn et al., 2019; Cuevas et al., 2018a; Prom et al., 2019), and it is not surprising to find another zinc finger related SNP as a top candidate for sorghum defense to anthracnose here.

The closest annotated coding region from the SNP locus S05_64406687 is a sulfotransferase. The sulfotransferase family of enzymes play critical roles in plant develop and stress responses, including host defense (Hirschmann et al., 2014). In sorghum, sulfotransferase has been implicated to be related to susceptibility or resistance to the parasitic weed striga based on a change in the structure of a compound in the root exudate so that it no longer stimulates seed germination (Gobena et al., 2017). The SNP locus S05_64406687 as a top candidate in response to *C. sublineola* might indicate that this sulfotransferase is also related to biotic stress in sorghum.

The SNP locus S08_61651261 tags DUF594 and DUF4220, related genes that are both listed as having unknown function. However, when their motifs were examined using Prosite, they were found to include a thaumatin‐like family profile, and thaumatins are broadly recognized as PR‐5 (pathogen‐related protein 5) having a role in disease resistance (de Jesús‐Pires et al., 2020). Induction of expression of a gene encoding a transmembrane protein with a DUF594 domain was found in maize root inoculated with *C. graminicola*, and this can be related to fungal recognition (Miranda et al., 2017). Similarly, in another study, it had been speculated that DUF4220 is a resistance‐related gene in *Brachypodium distachyon* to the wheat stripe rust pathogen *Puccinia striiformis* f. sp. *tritici* (Gilbert et al., 2018).

The SNP locus S03_53329242 is very near a gene that encodes an aldehyde dehydrogenase. Although it is not reported specifically in sorghum, pepper aldehyde dehydrogenase CaALDH1 interacts with *Xanthomonas* type III effector AvrBsT and promotes effector‐triggered cell death and defense responses (Kim & Hwang, 2015).

The SNP locus S02_65282291 is within a single strand DNA repair‐like protein. The DNA repair proteins are known to be directly involved in regulation of gene expression during plant immune response (Song et al., 2011).

Finally, the closest annotated gene of S03_10745341 has an F‐box domain. The use of virus induced gene silencing of an F‐box gene was found to increase susceptibility of barley to downy mildew (Dagdas et al., 2009) and similar studies have shown F‐box proteins are required for the regulation of cell death involved in hypersensitive resistance in tomato and tobacco (*Nicotiana tabacum* L.) (van den Burg et al., 2008). Thus, as in the case of leucine‐rich repeat and zinc finger proteins, the multiple appearances in sorghum GWAS studies of F‐box related genes enhances the prospect they play important roles in disease response to *C. sublineola* (Ahn et al., 2019; Cuevas et al. 2018a).

Testing the genes identified by SNPs for associated levels of mRNA is a future target of research through real‐time quantitative reverse transcription PCR (Real‐time qRT‐PCR) or RNA sequencing analysis (RNA‐Seq). Some SNPs, related to leucine‐rich repeat, zinc finger, and F‐box related proteins, have already been reported as top GWAS‐identified candidates for sorghum defense against *C. sublineola* in other collections of sorghum cultivars. The Senegalese sorghum lines have not been extensively studied for responses to anthracnose, so GWAS for response to *C. sublineola* provided possible sources of sorghum defense to anthracnose. Although the lines were newly studied for anthracnose response and GWAS, these proteins identified in other collections are again listed as top candidates, which is a strong implication of the genes’ importance in sorghum defense. In addition, candidate genes coding for these proteins have been reported as important defense‐related genes in other plants.

None of the SNP passed Bonferroni adjustments with *p* < .05; however, Bonferroni adjustments is often considered too conservative (Perneger, 1998). Hence, a *p* value less than .0003 cutoff was applied in this study, and all eight top candidate genes had a known association with pathogen defense. Revisited top candidate genes provides strong evidence for their role in defense against *C. sublineola* across different sorghum populations. On the other hand, the newly detected SNPs in sorghum are expected to provide more options to explore for anthracnose resistance in the future. Furthermore, the current trial in Texas and the study conducted in Puerto Rico by Cuevas et al. (2018b) noted that the Senegalese sorghum collection maintained by NPGS is a good source for anthracnose resistance germplasm.

## AUTHOR CONTRIBUTIONS

Ezekiel Ahn: Conceptualization; Formal analysis; Validation; Writing‐original draft. Louis K. Prom: Data curation, Investigation; Methodology; Resources; Validation; Visualization; Writing‐review & editing. Zhenbin Hu: Formal analysis; Software; Validation; Writing‐review & editing. Gary Odvody: Methodology; Resources. Clint Magill: Funding acquisition; Project administration; Supervision; Writing‐review & editing.

## CONFLICT OF INTEREST

The authors declare that they have no conflicts of interest.

## Supporting information

Supplemental table 1 lists the accessions obtained from the USDA‐ARS, Plant Genetic Resources Conservation Unit for this research, and raw phenotypic data.

## Data Availability

The raw phenotypic data is available as a Supplemental Table S1. Genotypic data was extracted from an integrated sorghum SNPs dataset based on sorghum reference genome version 3.1.1 and originally genotyped using GBS (Elshire et al., 2011; Hu et al., 2019; Upadhyaya et al., 2013; Wang et al., 2013). The top SNP alleles scored, positions in the context of a reference sequence, and the *p* value for each trait and each SNP are discussed in the main text.
